# Capture-SELEX: Selection Strategy, Aptamer Identification, and Biosensing Application

**DOI:** 10.3390/bios12121142

**Published:** 2022-12-07

**Authors:** Sin Yu Lam, Hill Lam Lau, Chun Kit Kwok

**Affiliations:** 1 State Key Laboratory of Marine Pollution and Department of Chemistry, City University of Hong Kong, Hong Kong SAR 999077, China; 2 Shenzhen Research Institute, City University of Hong Kong, Shenzhen 518057, China

**Keywords:** nucleic acid aptamer, DNA and RNA Capture-SELEX, small molecule contaminant, characterization, aptamer, biosensing applications

## Abstract

Small-molecule contaminants, such as antibiotics, pesticides, and plasticizers, have emerged as one of the substances most detrimental to human health and the environment. Therefore, it is crucial to develop low-cost, user-friendly, and portable biosensors capable of rapidly detecting these contaminants. Antibodies have traditionally been used as biorecognition elements. However, aptamers have recently been applied as biorecognition elements in aptamer-based biosensors, also known as aptasensors. The systematic evolution of ligands by exponential enrichment (SELEX) is an in vitro technique used to generate aptamers that bind their targets with high affinity and specificity. Over the past decade, a modified SELEX method known as Capture-SELEX has been widely used to generate DNA or RNA aptamers that bind small molecules. In this review, we summarize the recent strategies used for Capture-SELEX, describe the methods commonly used for detecting and characterizing small-molecule–aptamer interactions, and discuss the development of aptamer-based biosensors for various applications. We also discuss the challenges of the Capture-SELEX platform and biosensor development and the possibilities for their future application.

## 1. Introduction

Accumulating evidence suggests that small-molecule contaminants, such as antibiotics, pesticides, and plasticizers, pose a severe threat to human health and the environment worldwide. Antibiotics have been extensively used for decades to treat bacterial infections, and most are recalcitrant to biodegradation and elimination [1]. Moreover, the global overuse of antibiotics has led to their long-term persistence in the marine environment. More than 700,000 human deaths per year are attributable to bacterial resistance to antimicrobials (including antibiotics), with this number projected to increase to 10 million by 2050 [2]. Pesticides are widely used in agriculture to control pests and disease carriers. According to the World Health Organization, 193,460 unintentional deaths in 2012 were due to pesticide poisoning [3]. Plasticizers are widely applied in plastic products, such as Di(2-ethylhexyl)phthalate (DEHP), contaminating the environment. Toxic plasticizers with their toxicological effects, including endocrine disruption, carcinogenicity, and bioaccumulation potential [4,5,6], may enter human body via inhalation, ingestion, and dermal contact, reflecting the high risk of harm to human health.

Some traditional techniques for detecting small-molecule contaminants with high accuracy include high-performance liquid chromatography [7,8], enzyme-linked immunosorbent assays [9,10], and liquid chromatography–mass spectrometry [11,12]. However, these techniques are costly and laborious, and can only be performed by professional technicians. Therefore, there is an urgent need for robust aptamer-based biosensors (also called aptasensors), for low-cost, user-friendly, and rapid detection of small-molecule contaminants.

Aptamers are short, single-stranded DNA or RNA oligonucleotides that are able to bind its target with high affinity and specificity. The development of an aptasensor for detecting small-molecule contaminants requires the generation of a novel aptamer. It has gradually replaced traditional antibodies in some applications as they have several advantages over antibodies, such as thermal stability, low cost, amenability to chemical modification, and ease of generation [13,14]. Furthermore, when designed appropriately, the chemical modification of aptamers often does not affect—and sometimes even strengthens—their binding affinity for their targets, whereas the chemical modification of antibodies may remove their affinity for their targets [13]. Moreover, antibodies can only bind to a narrow range of targets, such as immunogenic non-toxic molecules and other molecules that do not cause strong immune responses [13]. In contrast, aptamers can selectively and tightly bind to a broad range of targets, including proteins [15,16,17], cells [18,19], microorganisms [20], and small-molecule contaminants [21,22,23,24], which may be either immunogenic or non-immunogenic. In addition, the in vivo generation of antibodies is a lengthy process (~6 months or longer) and requires a living host, whereas aptamers can be generated through in vitro selection (~2–8 weeks) using a method known as systematic evolution of ligands by exponential enrichment (SELEX) [13].

**Figure 1:**
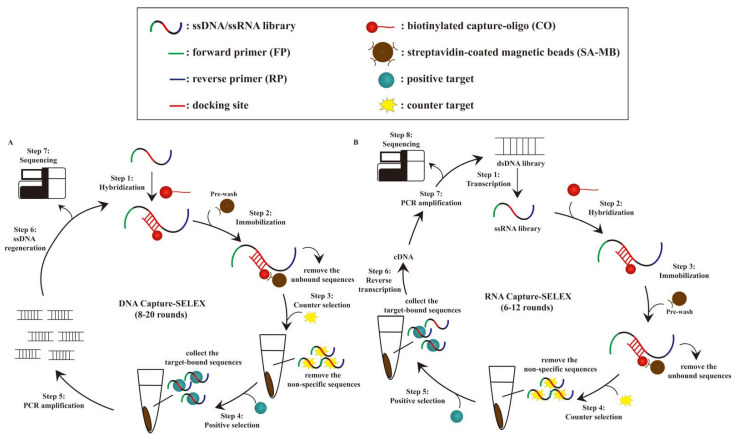
The experimental flowchart of DNA Capture-SELEX and RNA Capture-SELEX. (**A**) DNA Capture-SELEX. Each DNA Capture-SELEX selection round consists of 7 main steps: (1) The designed ssDNA library template is hybridized with biotinylated capture-oligonucleotide. (2) The hybridized product is immobilized on the washed streptavidin-coated magnetic beads. (3–4) Target-bound sequence elution: counter selection is followed by positive selection. (5) PCR amplification was performed on the eluted ssDNA to obtain the amplified dsDNA pool, (6) and regenerate ssDNA for the next DNA Capture-SELEX round (either gel purification or magnetic bead-based method). (7) After DNA Capture-SELEX, aptamer sequences were identified through sequencing. (**B**) RNA Capture-SELEX. The principles and procedures are similar to (**A**), except that RNA Capture-SELEX consists of additional transcription (step 1) to convert DNA to RNA and reverse transcription (step 6) to convert RNA to DNA steps.

Three research groups independently developed the first SELEX method and all reported their findings in 1990: Tuerk and Gold [25], Ellington and Szostak [26], and Joyce [27]. Since then, many derivative methods of SELEX have been developed, such as capillary electrophoresis (CE)-SELEX [28,29,30], graphene oxide (GO)-SELEX [31,32,33], cell-SELEX [34,35,36], in vivo SELEX [37,38], and Capture-SELEX [22,23,39]. Capture-SELEX selects DNA or RNA aptamers that bind small-molecule contaminants and is partly based on FluMag-SELEX. Stoltenburg et al. reported FluMag-SELEX in 2005 [40], and it is based on the magnetic bead (MB)-based SELEX, whereby the fluorescein-labeled oligonucleotides is to monitor the enrichment of target-specific aptamers with the quantification in each round, and biotinylated target molecules are immobilized onto the surface of streptavidin-coated magnetic beads (SA-MBs). However, it is more challenging to immobilize biotinylated small-molecule targets than biotinylated large-molecule targets such as proteins [41], as small-molecule targets have fewer functional groups. Thus, Capture-SELEX is an effective alternative to FluMag-SELEX, as Capture-SELEX involves the immobilization of a biotinylated hybridized library on a solid support, which is more feasible than the immobilization of biotinylated targets on a solid support (Figure 1). Due to the differences in the principle, Capture-SELEX also has some deficiencies compared to other SELEX methods [41]. The false-positive may happen in the immobilization process of Capture-SELEX. The complemented library may have a dynamic dissociation equilibrium due to the weak force, and some unbound sequences (non-biotinylated) are also dissociated from the solid support. Moreover, conformational changes of the target are essential for the success of aptamer screening and diversity in the selection process. This review discusses Capture-SELEX-based selection strategies for small-molecule contaminant targets, methods used to identify and characterize small molecule–aptamer interactions, and biosensing applications of aptamers generated using Capture-SELEX.

## 2. Principle and General Procedure of Capture-SELEX for Aptamer Selection

### 2.1. Overview of DNA and RNA Capture-SELEX

The classical procedure of the DNA Capture-SELEX selection method involves hybridization, immobilization, target-bound sequence elution, polymerase chain reaction (PCR) amplification, regeneration of single-stranded DNA (ssDNA), and sequencing (Figure 1A). Briefly, in the first step, a random ssDNA library is designed with a docking site that is complementary to the biotinylated capture oligonucleotides, and these ssDNAs are hybridized with capture-oligonucleotides to form biotinylated hybridized library via Watson-Crick base pairing. Next, the biotinylated library is immobilized onto SA-MBs, which function as solid support, and the loaded SA-MBs are then washed several times to remove the residual and non-specific sequences using a magnetic rack and generate a washed biotinylated library on the MBs (which comprise superparamagnetic iron oxide). Subsequently, a magnetic rack also used to discard supernatant (unbound or non-specific sequences), after which the counter-selection process is performed, wherein a counter target with a similar chemical structure and concentration as the positive targets, enhances the binding affinity and specificity of aptamers towards it positive small-molecule target [42]. Counter selection can be performed either in the last few rounds [39,43] or between the selected rounds [44,45] of Capture-SELEX, and the concentration of the counter target should be the same as the positive target in step 3 (Figure 1A). The negative selection is followed by positive selection. Next, an elution is performed; if the binding affinity between the target and aptamer is higher than that between the aptamer and the capture oligonucleotide, the target-bound aptamer candidates are eluted. As the library sequences interact with the positive targets, only sequences with conformational changes due to the interaction can be eluted from the MBs [46].

In this review, we summarize two main strategies used to regenerate ssDNA from amplified dsDNA in step 6 (Figure 1A): the gel purification method [21,23,47] and the MB-based method [24]. A primer is designed and modified in order to identify the sense strand in the denaturing polyacrylamide gel, such as a reverse primer with poly-dA_20_ extension or a forward primer labeled with either fluorescein or the cyanine dye Cy3. The gel purification method involves denaturing polyacrylamide gel electrophoresis (PAGE), followed by extraction and purification. The MB-based method begins with PCR products with biotinylated reverse primers being incubated to facilitate their deposition onto MBs. The supernatant is then removed, and the non-biotinylated forward strands of dsDNA PCR products are released from the MBs by treatment with sodium hydroxide and then neutralized by treatment with an appropriate amount of tris(hydroxymethyl)aminomethane hydrochloride or hydrochloric acid. The regenerated ssDNA in each round is collected for the next round of selection and then prepared for sequencing after several rounds of selection. Currently, Capture-SELEX is mostly applied for the selection of DNA aptamers rather than RNA aptamers. RNA Capture-SELEX adopts the same selection strategies as DNA Capture-SELEX, except for including two more steps: transcription and reverse transcription (Figure 1). In addition, more rounds need to be performed in DNA Capture-SELEX (8–20 rounds) than in RNA DNA Capture-SELEX (6–12 rounds) to enrich the target-bound pool sufficiently.

### 2.2. Sanger and Next-Generation Sequencing and Analysis

Sequencing is a crucial step performed after the SELEX-based selection of aptamer candidates and before aptamer–target characterization. Here, we review two of the main methods used for sequencing aptamers, those being Sanger sequencing and next-generation sequencing (NGS). Sanger sequencing is a DNA sequencing technology based on the chain termination method invented by Frederick Sanger and his colleagues [48] in 1997. The main workflow involves blocking the polymerase-mediated elongation of DNA through the incorporation of fluorophore-labeled dideoxynucleotides (dideoxyadenosine triphosphate, dideoxyguanine triphosphate, and dideoxythymine triphosphate) at the 3′ ends of DNA sequences, resulting in various lengths of DNA fragments for size separation and fluorescent-based detection. This method has the advantages of high precision, high efficiency, and low radioactivity, and the disadvantages of being expensive and having low-quality primer binding in the first 15 to 40 base pairs [49]. Subsequently, high-throughput second- and third-generation sequencing were invented and superseded Sanger sequencing [50]. In 1998, Balasubramanian and Klenerman co-invented Solexa sequencing [51], also known as NGS. This method differs from Sanger sequencing mainly in its mode of chain termination: modified deoxynucleoside triphosphates (dNTPs) with a reversible terminator are used to terminate polymerization and are then removed to allow incorporation of the next modified dNTP [52]. NGS offers some advantages over Sanger sequencing, including enabling massively parallel sequencing in a short period and having a lower cost per base pair.

In order to analyze sequencing data, in silico technique towards SELEX facilitate the aptamer selection [53,54,55]. The initial step is primary sequence analysis using some bioinformatic tools, such as ClustalW [56] and Clustal Omega (https://www.ebi.ac.uk/Tools/msa/clustalo/ (accessed on 12 October 2022)) [57]. Multiple sequence alignment is performed to divide sequences into families or clusters. The conserved regions of the sequences are analyzed using Gblocks software. Furthermore, Multiple Expectation maximizations for Motif Elicitation (https://meme-suite.org/meme/tools/meme (accessed on 12 October 2022)) [58] is used to identify the sequence motif, which provides the basis for downstream analysis. Aptamers can form diverse secondary structures, such as stem-loop, triplex, G-quadruplex, and pseudoknot structures; thus, enriched sequences can also be selected based on secondary structure and Gibbs free energies (ΔG) prediction. Several nucleic acid structure-prediction Web servers are available and are often used to computationally predict secondary structures and Gibbs free energies (ΔG) of aptamer sequences. These include MFOLD (http://www.unafold.org/mfold/applications/dna-folding-form.php (accessed on 12 October 2022)) [59], KineFold (http://kinefold.curie.fr/ (accessed on 12 October 2022)) [60], and RNAstructure (https://rna.urmc.rochester.edu/RNAstructure.html (accessed on 12 October 2022)) [61]. The predicted secondary structures were then converted into unique three-dimensional (3D) structures using online web servers. These include fragment-based methods: RNAComposer (https://rnacomposer.cs.put.poznan.pl/ (accessed on 12 October 2022)), 3dRNA (http://biophy.hust.edu.cn/3dRNA (accessed on 12 October 2022)), and Vfold 3D (http://rna.physics.missouri.edu/vfold3D/ (accessed on 12 October 2022)); and energy-based method: simRNA (https://genesilico.pl/SimRNAweb (accessed on 12 October 2022)) [62]. Molecular docking tools include AutoDock, AutoDock Vina, and DOCK, which were used to predict the predominant binding modes and regions of the target molecule based on the generated binding scores for specific sequences [63,64,65]. After a few prediction steps, the aptamer candidates are shortlisted and subjected to binding tests and characterization.

## 3. Small-Molecule–Aptamer Interaction and Characterization

**Figure 2:**
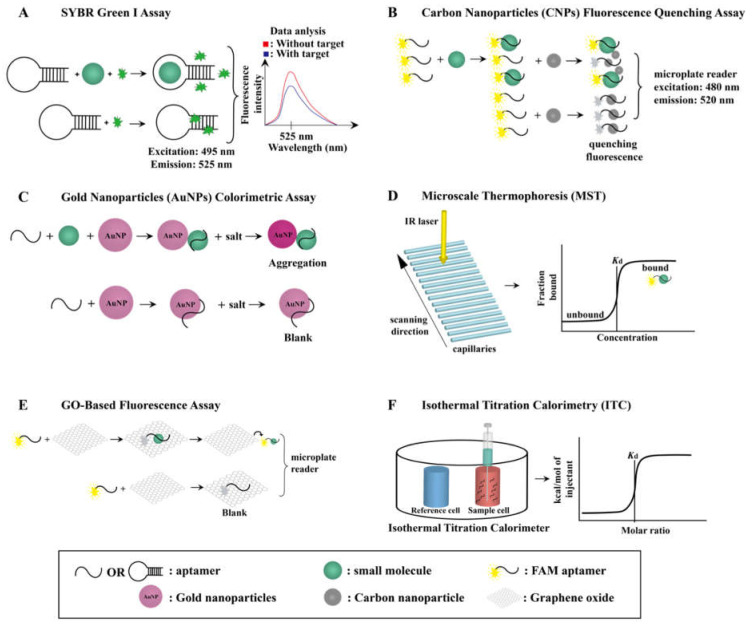
A schematic illustration of six commonly used methods for detecting interactions between small molecule and aptamer. (**A**) SYBR green I (SGI) assay. (**B**) Carbon nanoparticles (CNPs) fluorescence quenching assay. (**C**) Gold nanoparticles (AuNPs) colorimetric assay. (**D**) Microscale thermophoresis (MST) assay. (**E**) Graphene oxide (GO)-based fluorescent assay. (**F**) Isothermal titration calorimetry (ITC) assay.

The secondary structures of aptamers often organize into complex three-dimensional structures to maintain global stability and form functional conformations. Therefore, a stable aptamer can strongly bind to small molecules and shape the aptamers-target complex via weak noncovalent interaction forces, including hydrogen bonding, π-π stacking, van der Waals forces, hydrophobic, and electrostatic interactions. The aptamer-target interactions may either depend on the availability of narrow binding pockets in the three-folded structures of aptamers or the chemical characteristics of targets and the aptamer candidate or the length of the oligonucleotides, for example, removing excess flanking nucleotides outside the binding site (truncated aptamer), which is also a strategy to improve binding affinity and specificity [66,67]. In order to assess the binding strength and specificity of the interaction between an aptamer and its target, binding assays are used to determine the dissociation constant (*K*_d_) (Table 1), i.e., the binding affinity between an aptamer and its cognate target (and between an aptamer and its non-cognate targets), where the lower the *K*_d_ value, the stronger the interaction. Several factors may influence the *K*_d_ value, such as temperature, pH value, ionic concentrations, and hydrophobicity of the solution [66]. Below, we discuss the six standard assays used for characterizing the interactions between small-molecule contaminants and aptamers generated using DNA/RNA Capture-SELEX (Figure 2). In contrast with traditional methods, such as HPLC and LC-MS, these assays are highly sensitive, non-toxic, and inexpensive.

### 3.1. SYBR Green I (SGI) Assay

The binding affinity of Capture-SELEX-generated aptamers for small molecules can be identified by a novel label-free fluorescence SGI assay [24,68,70,73,83]. SGI is a fluorescent nucleic-acid-intercalating dye that was introduced in the 1990s and is widely applied in real-time PCR [92,93,94], fluorescent gel imaging [95,96], and flow cytometry [97,98]. It intercalates into the minor groove of DNA base pairs or adenine–thymine-rich stem-loop sites to form a fluorescent complex with DNA [99]. The fluorescence intensity of SGI–DNA complexes is measured in the range of 505 to 650 nm, with an excitation wavelength of 495 nm and an emission wavelength of 525 nm, using a spectrophotometer or a microplate reader; thus, an SGI assay identifies the binding affinity of aptamers for small-molecule targets by detecting the change in the fluorescent signal induced by the dissociation of SGI from the aptamers upon their binding with these small-molecule targets, and therefore the fluorescence intensity decreases (Figure 2A). The fluorescence intensity will be recovered as the concentration of the aptamers’ small-molecule targets increases.

### 3.2. Carbon Nanoparticle (CNP) Fluorescence Quenching Assay

CNPs can be readily synthesized [45] in research laboratories, have low toxicity, and are biocompatible [100]. Due to the strong fluorescence-quenching ability of CNPs’ sp^2^- and π-rich structures, they are useful for characterizing the binding affinity of aptamers for small molecules. This means that when an aptamer is adsorbed onto the surface of CNPs, π- π stacking interactions that form between the nucleobases and nucleosides of the aptamer and the CNPs result in fluorescence quenching. Therefore, in the absence of aptamers’ small-molecule targets, aptamers are bound to CNPs, resulting in fluorescence quenching, whereas in the presence of aptamers’ small-molecule targets, aptamers are bound to these targets rather than CNPs, and thus fluorescence is not quenched (Figure 2B) [101].

### 3.3. Gold Nanoparticle (AuNP) Colorimetric Assay

The AuNP colorimetric assay is widely used for characterizing the binding affinity of an aptamer for its target [24,45,70,71,76,80,83,84]. It is a label-free assay as AuNPs allow the identification of a target reaction based on colorimetric signals. AuNPs can be synthesized by reducing chloroauric acid with citric acid and ascorbic acid (the Turkevich method) [102] or with sodium citrate [103]. The Turkevich method affords AuNPs coated with negatively charged citrate ions, and thus the AuNPs are treated with sodium chloride to neutralize their surface charge and thereby induce their aggregation (Figure 2C) [104]. Owing to the electrostatic screening effect, the surface plasma resonance absorption peak of AuNPs undergoes a red shift from 520 nm to 650 nm, which is measured using a microplate reader and reflects the binding affinity of aptamers to their small-molecule targets. In the absence of their small-molecule target, aptamers are adsorbed onto the surface of AuNPs, increasing their colloidal stability of AuNPs and reducing their salt-induced aggregation [105]. However, in the presence of their small-molecule targets, aptamers binds to their targets rather than AuNPs, which decreases the colloidal stability of AuNPs and thereby increases their salt-induced aggregation. This results in a solution containing AuNPs, aptamers, and their small-molecule targets changing color from red to pink to purple or even to blue or gray [70].

### 3.4. Microscale Thermophoresis (MST) Assay

The MST assay is a powerful tool for testing the binding affinity of aptamers for their small-molecule targets as it only requires a small amount of sample (up to 4 μL), involves a simple preparation process, and rapidly provides accurate results (within 15 min) [75,76,80]. It is a fluorophore-labeled and immobilization-free assay that is carried out using a Monolith NT.115 instrument. As shown in Figure 2D, the capillary tray of this instrument can accommodate up to 16 thin glass capillaries for operation. An infrared laser heats a specific capillaries area, creating a microscopic temperature gradient on them. Thus, when an aptamer binds to its small-molecule target, there is a significant change in fluorescence intensity due to thermophoresis. An MST binding curve (dose-response curve) is plotted using Affinity Analysis Software to represent specific binding that is specific to its small-molecule target, which automatically calculates the *K*_d_ of an aptamer for its small-molecule target.

### 3.5. GO-Based Fluorescent Assay

GO can be synthesized from graphite via the Hummers and Offeman method [106], which uses potassium permanganate (as the oxidizing agent) and sodium nitrate in a solution of sulfuric acid [107]. GO has an sp^2^ structure and numerous oxygen-containing functional groups [108], such as carbonyl groups, hydroxyl groups, and carboxylic acid groups, and thus can strongly quench fluorescence. This quenching occurs when a 5′-fluorescein-labeled aptamer is adsorbed onto the surface of GO through π- π stacking interactions and hydrogen bonding (Figure 2E). When 5′-fluorescein labeled aptamer is absorbed onto GO, GO-mediated fluorescence quenching occurs. The fluorescence intensity will be recovered when the labeled aptamer is desorbed from the surface of GO in the presence of small-molecule target. The fluorescence intensity change are measured using a microplate reader, and eventually the *K*_d_ value of the aptamer for its small-molecule target is calculated [72,77,109].

### 3.6. Isothermal Titration Calorimetry (ITC) Assay

The ITC assay is a label-free and immobilization-free assay for measuring the binding affinity of an aptamer for its small-molecule target [44,83] in an instrument comprising a reference cell and a sample cell (Figure 2F). The reference cell is filled with a buffer and water (without the aptamer or its small-molecule target), while the sample cell is filled with the buffer and the aptamer and is titrated via syringe with up to 20 volumes of the aptamer’s small-molecule target [77]. The temperature in the two cell units is maintained at a constant value, and when the small molecule is titrated against the aptamer in the sample cell, an exothermic binding reaction occurs. The feedback system of the ITC instrument then reduces the power supply to the sample cell to prevent an increase in temperature, leading to a difference in the amount of energy supplied to the reference cell and the sample cell. Therefore, during this titration, the power supply to the sample cell continuously decreases until the small-molecule–aptamer binding has been completed. The binding affinity of the aptamer can then be determined from the energy curve generated [110]. The ITC instrument measures the exothermic reaction (heat released) and endothermic reaction (heat absorbed) when the molecules interact. The peak goes downward when the exothermic reaction occurs, whereas an upward peak is measured when an endothermic reaction occurs. The downward peak between the interaction of aptamer and small-molecule target are measured from the ITC instrument using software such as MicroCal ITC200 and MicroCal PEAQ-ITC.

## 4. Development of Aptamer-Based Biosensors

**Figure 3:**
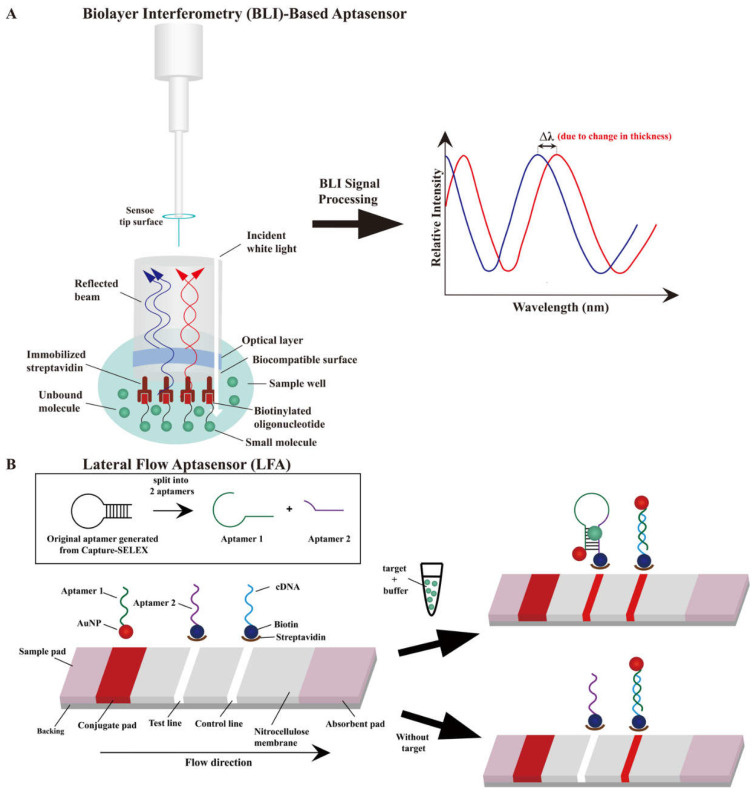
The schematic illustration of aptasensors for the detection of small molecules using novel aptamer generated from Capture-SELEX. (**A**) Biolayer interferometry (BLI)-based aptasensor. (**B**) A lateral flow aptasensor (LFA) is generally constructed by 4 sections: sample pad, conjugate pad, nitrocellulose membrane with test line and control line, and absorbent pad. The sample flow from left to right laterally (left). When the target is loaded onto the sample pad, positive result (red band for both control and test line) is observed after 15 min (right).

To date, Capture-SELEX has been used for screening and generating a series of aptamers against small-molecule contaminants (Table 1). As aptasensors can be used for low-cost, rapid, and real-time detection of small-molecule contaminants, they have increasingly supplanted conventional antibody-based biosensors. Fluorescent aptasensors are the most popular (Table 2), and their working principle is similar to that of fluorescent-based characterization methods. Therefore, two other types of aptasensor are discussed here (Figure 3).

### 4.1. Biolayer Interferometry (BLI)-Based Aptasensor

Optical-fiber-based BLI biosensors have been developed for real-time, sensitive, and rapid measurement [112] of interactions between biomolecules, including DNA–protein [113,114], antibody–antigen [115], and DNA–small-molecule-contaminant interactions [116,117]. The sensor tip of an optical fiber consists of two reflective surfaces: a streptavidin-coated biocompatible surface, which is immersed in the sample solution, and an optical layer, which functions as an internal surface (Figure 3A). The biocompatible surface is functionalized to immobilize a biotinylated aptamer and minimize non-specific binding. Inside the sensor, these two surfaces generate an interference pattern by reflecting incident white light. When an aptamer binds to its small-molecule target, a spectral redshift (Δλ) occurs as the bound compound immobilizes on the tip surface and the surface thickness increases. The false-positive results from this sensor can be minimized as the non-specific and unbound molecules can be differentiated from the molecules with high binding affinity, resulting in high detection accuracy [116].

### 4.2. Lateral Flow Aptasensors (LFAs)

LFAs have been developed as portable detection devices with cost-effective, rapid (≤15 min), and easy operation and broad applications, such as in the detection of pregnancy, severe acute respiratory syndrome coronavirus 2 [118,119], and infectious diseases [120]. Owing to the drawbacks of antibodies, LFAs are gradually replacing antibody-based lateral flow biosensors, although the latter remains predominantly used. In addition, LFAs have been used to detect antibody-inaccessible small-molecule contaminants [24,89,121]. Figure 3B depicts the LFA concept [121,122,123], in which AuNPs are typically used for colorimetric identification of the test line and control line on the strip [124]. The high affinity aptamer generated from Capture-SELEX against the specific small-molecule target that splits into two fragmented aptamers (aptamer 1 and 2; Figure 3B) [123]. A sample solution (a solution of an aptamer’s small-molecule target in buffer) is loaded onto the sample pad and flows through the conjugate pad (containing AuNP–aptamer 1 conjugates) by capillary action. When aptamer–small-molecule-target binding occurs, two fragmented aptamers could rejoint the three-dimensional structure without affecting the binding affinity, and thus a red band appears in the test line. A complementary DNA (cDNA) is designed to hybridize with aptamer 1 in the control line and generate a visual signal, which is used to verify whether the aptasensor is working properly based on the appearance of red band in both presence or absence of the aptamer target. The excess sample reagent flows from the test line to the control line and to the absorbent pad eventually. Two slightly different LFAs have also been developed that use aptamers generated using Capture-SELEX. The LFA devised by Du et al. [24] combines lateral flow strips and the recombinase polymerase amplification technique for detecting erythromycin in tap water, whereas that devised by Xia et al. [89] uses aptamer EC1-34 as a recognition probe for detecting ethyl carbamate. The latter LFA differs from the former LFA only in terms of its use of a cationic polymer (such as poly(dimethyldiallylammonium chloride)) instead of streptavidin in the test line.

## 5. Biosensor Applications

### 5.1. Food Safety Analysis

#### 5.1.1. Veterinary Drug Residues and Pesticides

Ractopamine (RAC) is a β-adrenergic agonist used illegally as an animal feed additive for increasing skeletal muscle mass, reducing fat deposition, and increasing protein accretion in livestock [125,126]. The accumulation of RAC in animals may increase the risk of food poisoning in humans and have other adverse effects on human health, such as causing headache, tachycardia, and muscle tremors [127]. Therefore, the development of a rapid and cost-effective biosensor for RAC contamination in food is warranted. Duan et al. [82] obtained nine aptamer candidates for RAC through 16 selection rounds of Capture-SELEX. The aptamer RAC-6 showed the highest binding affinity for RAC in a GO-based fluorescent assay, with weak binding affinity for other off-target species (<22%). The researchers further developed a fluorescent aptasensor based on RAC-6 that exhibited a linear detection range of 0.33 to 331.79 nM, a low limit of detection (LOD; 0.13 nM; Table 2), and high recovery rates (82.57–104.65%). They found that RAC-6 was especially useful for detecting RAC contamination in pork samples.

λ-Cyhalothrin is a broad-spectrum pyrethroid insecticide used to control agricultural insect pests, such as Coleoptera, Lepidoptera, and mites, and thus increase agricultural productivity [128]. Compared with older-generation pesticides, the insecticidal effect of λ-cyhalothrin is 10–100 times stronger, and overuse of this insecticide may lead to food contamination [129]. Due to its toxicity, the ingestion of λ-cyhalothrin residues in food may cause serious adverse effects, including mouth ulcers, nausea, abdominal pain, and vomiting [130]. Yang et al. [76] used Capture-SELEX to obtain several candidate aptamers against λ-cyhalothrin. The aptamer LCT-1 showed the strongest affinity and specificity for λ-cyhalothrin in a colorimetric assay and its binding affinity was further optimized by truncation. The dissociation constant of the truncated aptamer, named LCT-1-39, was improved by approximately 40 nM relative to LCT-1, and similar results were obtained using the MST assay. LCT-1 and LCT-1-39 were thus used to establish colorimetric aptasensors for detecting λ-cyhalothrin. These aptasensors demonstrated low LODs for LCT-1 and LCT-1-39 (43.8 nM and 41.35 nM, respectively; Table 2) and mean λ-cyhalothrin recovery rates of 82.93–95.50%. Compared with traditional quantification methods, these colorimetric aptasensors demonstrated more rapid detection of λ-cyhalothrin in cucumber and pear samples.

#### 5.1.2. Food Additives and Flavoring Agents

Vanillin is the second most popular flavoring agent worldwide and is used as a food additive in sweet foods and beverages, and a masking agent in numerous pharmaceutical formulations [131]. It is a phenolic aldehyde that has demonstrated antioxidant, antimicrobial, and antifungal activities in various food products [132,133]. Hence, a rapid detection biosensor is required to monitor vanillin concentrations during processed food production. Through Capture-SELEX, Kuznetsov et al. [23] obtained six aptamer candidates against vanillin and found that Van_74 had the highest binding affinity by nondenaturing PAGE. Its specificity for vanillin was also confirmed in the presence of interferents, such as benzaldehyde, guaiacol, furaneol, ethyl guaiacol, and ethyl vanillin. The authors also found that Van_74 was sensitive to the composition of the selection buffer. Van_74 was then used in the development of an ion-sensitive field-effect transistor (ISFET)-based biosensor that demonstrated a low LOD (0.155 μM) and a dynamic detection range of 0.155–1 μM (Table 2). This novel aptasensor can be applied for the rapid on-site detection of vanillin contamination in coffee extracts and mixtures.

Another aroma compound, furaneol, is extensively used as an artificial flavoring agent as it imparts fruit flavor to food [134], and thus a rapid detection biosensor is required to monitor furaneol concentrations during processed food production. Komarova et al. [68] obtained eight aptamer candidates against furaneol through 13 selection rounds of Capture-SELEX. These aptamers’ binding affinity for furaneol was analyzed by three methods: an exonuclease protection assay, an SGI assay, and an MB-associated elution assay. The results revealed that the aptamer Fur_14 had the highest binding affinity for furaneol; therefore, Fur_14 was used to develop an ISFET-based aptasensor. Fur_14 was further modified with an alkyne label at its 5′-end, and this Fur_14 derivative exhibited a furaneol detection range of 0.1–10 μM (Table 2).

Spermine, tyramine (TYR), and β-phenethylamine (PHE) are biogenic amines (BAs) that are typically present in foodstuffs. As the consumption of foods containing high concentrations of BAs may cause toxic effects, biosensors are needed for BA detection in foods [135,136]. Tian et al. [44] obtained aptamer candidates against spermine by Capture-SELEX selection and tested them using ITC and fluorescence assays. The aptamer APJ-6 showed the highest affinity and specificity for spermine and was, thus, used to develop a fluorescent aptasensor for spermine detection in pork samples. This aptasensor demonstrated a linear detection range of 0.1–20 nM and a low LOD (0.052 nM). For detecting TYR and PHE, Kuznetsov et al. selected and isolated several aptamers using Capture-SELEX. The selection process was monitored by the melting temperature (T_m_) in the screening process, and T_m_ peaked during the 14th round for both TYR and PHE. The aptamers TYR-2 and PHE-2 were identified to have the strongest binding affinity and specificity for TYR and PHE, respectively, based on a GO-based fluorescent assay. TYR-2 and PHE-2 were then used to develop fluorescent aptasensors for the detection of TYR and PHE in pork and bear meat samples. These aptasensors demonstrated LODs for TYR and PHE of 2.48 and 3.22 nM, respectively (Table 2), with target recovery rates in the range of 95.6–104.2%, suggesting their efficacy in detecting TYR and PHE in foods.

### 5.2. Aquatic Environment

#### 5.2.1. Veterinary Drug Residues and Pesticides

Erythromycin is a broad-spectrum macrolide antibiotic used to treat diseases such as diphtheria, pertussis, and bacillary angiomatosis [137]. The natural degradation of erythromycin is prolonged due to its stable structure, leading to increased erythromycin resistance among bacteria [138]. As erythromycin diffuses rapidly into most tissues of the human body, erythromycin pollution of environmental media poses a serious threat to human health, in addition to the ecosystem. Du et al. [24] obtained 10 aptamer candidates against erythromycin through 20 selection rounds of Capture-SELEX. The binding affinity and specificity of the candidates were determined using an SGI fluorometric assay, an AuNP-based colorimetric assay, a quartz crystal microbalance with dissipation assay, and an agarose chasing diffusion assay, resulting in the selection of the aptamer Ery_06 for the development of a novel LFA. This LFA demonstrated an erythromycin-detection range of 250–500 pM in water samples, with a low LOD (3 pM; Table 2) and rapid detection (within 15 min), suggesting its efficacy for erythromycin detection in water.

Roxithromycin is a macrolide antibiotic that poses a similar risk to the ecosystem and human health as erythromycin, indicating the need to establish a rapid and effective detection device for monitoring roxithromycin residues in environmental media. Jiang et al. selected aptamer candidates against roxithromycin after 16 selection rounds of Capture-SELEX. The aptamer Ap01 demonstrated the highest affinity and specificity for roxithromycin, as indicated by the results of an SGI assay, and was therefore selected for the development of a colorimetric aptasensor for roxithromycin. The developed aptasensor demonstrated a low LOD (0.077 μM) for roxithromycin in water samples (Table 2) and high recovery rates in the range of 90.48–109.39%.

#### 5.2.2. Toxins and Plasticizers

Gymnodimines (GYMs) are fast-acting cyclic imine toxins that are biosynthesized by dinoflagellates and have deleterious effects on the aquatic environment with the accumulation. The contaminated environment can have serious toxic effects on filter feeding shellfish and thereby pose a threat to human health [139]. Zhang et al. [75] used Capture-SELEX to screen and obtain six aptamer candidates against gymnodimine-A (GYM-A). G48 exhibited the highest binding affinity (*K*_d_: 288 nM) and was therefore chosen for further optimization and investigation. The truncated aptamer G48nop demonstrated an improved *K*_d_ value of 34.5 ± 1.72 nM and high specificity for GYM-A. A novel BLI-based aptasensor was established using this aptamer that detected GYM-A in the range of 55–1400 nM (linear range of 55–875 nM) and had a low LOD (6.21 nM; Table 2). This BLI-based aptasensor also demonstrated high recovery rates in the range of 96.65%–109.67%, indicating that is reliable and efficient in detecting and monitoring GYM-A in water samples.

Di(2-ethylhexyl) phthalate (DEHP) is a plasticizer that is widely used as an additive in packaging materials, and its residues are known to accumulate and dissolve in water [140]. DEHP is also a well-known endocrine disruptor that can enter the human body through ingestion of food or water and inhalation with contaminated air that disrupts the immune system. Lu et al. [71] selected aptamer candidates against DEHP through eight rounds of Capture-SELEX. Upon high-throughput sequencing and characterizing the candidate aptamers using an AuNP colorimetric assay and localized surface plasmon resonance, aptamer 31 was revealed to have high affinity and specificity. Aptamer 31 was thus used to develop an ultrasensitive electrochemical impedance spectroscopy aptasensor to detect DEHP in real water samples; this aptasensor demonstrated a low LOD (0.264 pM; Table 2) and a mean recovery rate ranging from 76.07% to 141.32%.

### 5.3. Other Potential Applications

Synthetic riboswitches can have several biotechnological applications, such as regulating gene expression, e.g., the construction of genetic circuits [141,142]. Natural riboswitches are mainly found in bacteria, while synthetic riboswitches are artificially generated by combining aptamer domains (using in vitro SELEX method) with expression platforms to regulate gene expression via small-molecule-RNA interactions [143,144]. However, using the conventional SELEX method, only a ciprofloxacin riboswitch aptamer has been developed, as most aptamers have limitations, such as excellent binding affinity and conformation switching, and require cellular screening after in vitro selection [145]. Subsequently, Boussebayle et al. [146] identified a paromomycin riboswitch aptamer using Capture-SELEX and found it had a high affinity (*K*_d_: 21 nM) using an ITC assay. Through further in vivo selection, this aptamer was revealed to have riboswitching properties. This work has introduced an efficient protocol for developing synthetic riboswitches and boosted the development of real-time intracellular biosensors for monitoring metabolic flows in living cells.

Zearalenone (ZEN) is a nonsteroidal estrogenic mycotoxin produced by fungi and is known to contaminate cereal grains and other crops [147]. Due to its high estrogenic activity, long-term intake of ZEN residues adversely affects human health by causing cervical cancer or hyperestrogenic syndrome [147,148]. Zhang et al. [84] obtained aptamer Z100 against ZEN after eight rounds of Capture-SELEX. Z100 was shown to have high affinity and specificity for ZEN in a fluorescence assay, and hence was selected to develop a rapid and on-site AuNP-based label-free aptasensor for detecting ZEN in agricultural produce. The developed aptasensor had a low LOD (12.5 nM), a linear detection range of 12.5–402.1 nM (Table 2), and high recovery rates in corn powder and feed (96.42–99.78% and 95.99–103.73%, respectively). This study revealed the great potential for developing aptamer-based inhibitors for ZEN to enhance animal feed safety.

Fenitrothion (FEN) is a broad-spectrum organophosphorus insecticide mainly used to control insect pests in agriculture [149]. As FEN is available at a low cost, large amounts of FEN are frequently applied in agriculture. High concentrations of FEN residues have been found in foods, which has become a great concern for human health and the environment. Trinh et al. [85] screened aptamer candidates against FEN through Capture-SELEX. In the thioflavin T (ThT) displacement assay, the aptamer FenA2 was identified to exhibit high-affinity FEN binding, as indicated by the loss of fluorescence. FenA2 was further optimized and used to develop a label-free ThT sensor. The developed aptasensor has a G4-quadruplex-like structure and a low LOD (14 nM; Table 2). This aptamer may be further optimized to develop a real-time FEN-detecting aptasensor.

## 6. Conclusions

Small-molecule contaminants are ubiquitous in the aquatic environment, agriculture produce, and animal feed due to the overuse of small molecules such as antibiotics and pesticides, and these contaminants pose a serious threat to human health and the environment. Owing to the few functional groups on these small molecules, screening aptamers against them is more challenging than doing so against large-molecule targets. Compared with other SELEX approaches, which involve the immobilization of small-molecule targets, the Capture-SELEX approach is more feasible, as they involve the immobilization of a biotinylated ssDNA/RNA library against which the binding affinity and specificity of small-molecule targets can be screened. To date, fewer than 50 studies have reported using Capture-SELEX to identify novel aptamers against specific small-molecule contaminants, suggesting that this process remains challenging, such as false-positive and lack of diversity. However, this research field has recently been receiving increasing attention from scientists. We hope that this review will encourage further research into the use of Capture-SELEX in generating aptamers against small-molecule contaminants. Six small-molecule aptamer characterization methods are introduced in this review. The high affinity and specificity aptamer work as a biorecognition element in aptasensor to detect specific small-molecule contaminants in environmental media and agricultural produce. This will help to improve food safety, aquatic environments, and agricultural crop production.

## Figures and Tables

**Table 1:** A list of small molecule contaminant-specific aptamers developed by Capture-SELEX method as of to date.

Name	Class	Aptamer Sequence (5′–3′)	nt	Dissociation Constant (*K*_d_)	Ref.
Kanamycin A CAS no.: 59-01-8 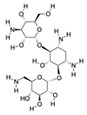	Aminoglycoside antibiotic	ATACCAGCTTATTCAATTAGCCCGGTATTGAGGTCGATCTCTTATCCTATGGCTTGTCCCCCATGGCTCGGTTATATCCAGATAGTAAGTGCAATCT	97	3.9 μM	[22]
Ofloxacin CAS no.: 82419-36-1 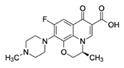	Antibiotic	ATACCAGCTTATTCAATTGCAGGGTATCTGAGGCTTGATCTACTAAATGTCGTGGGGCATTGCTATTGGCGTTGATACGTACAATCGTAATCAGTTAG	98	0.11 ± 0.06 nM	[39]
Furaneol CAS no.: 3658-77-3 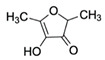	Aroma compound	CGACCAGCTCATTCCTCACCACGAGAAAGGAGCTCGATGAACTGCGAGCCGGATTCGACCCTATGCGAGTAGGTGGTGGATCCGAGCTCACCAGTC	96	1.1 ± 0.4 μM	[68]
Vanillin CAS no.: 121-33-5 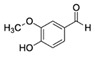	Flavoring	CGACCAGCTCATTCCTCAGGAGAAACATGGAGTCTCGATGATGTAGGAGCGGCGGAACGTAGGAAGAGAGGATGACGGAGGATCCGAGCTCACCAGTC	98	0.9 ± 0.3 μM	[23]
Erythromycin CAS no.: 114-07-8 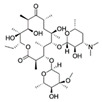	Antibiotic	AGGAATTCACGTCTCACTGGATTCACGCACGCCAAGGACTGCACTTAAGGTTAGATAGCCCCATGCAGTGAGTCAGGATATCG	83	20 ± 9 nM	[24]
Spermine CAS no.: 71-44-3 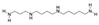	Biogenic amine	TATGAACGATTTACTCGTACAGACGACACTTATCATTTGC	40	9.648 ± 0.896 nM	[44]
Tetrodotoxin CAS no.: 4368-28-9 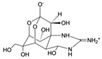	Toxin	ATACCAGCTTATTCAATTTAATGCGGGGTGAGGCTCAATCAAGGAAAGATATAAGTAAGCAAAAAGGTCAAACAAGGGCGAGATAGTAAGTGCAATCT	98	7 ± 1 nM	[43]
Chlorpromazine CAS no.: 50-53-3 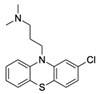	Phenothiazine	TCGGAGGGAAGTGCACCCATTCTTGGAAACAGGAGCTCCTGAACCGCCCACACGC	55	69.8 ± 9.81 nM	[69]
Roxithromycin CAS no.: 80214-83-1 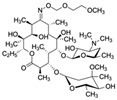	Antibiotic	ATTGGCACTCCACGCATAGGCACACCCACCGGCCTAGCCACACCATGCTGCTGTTGCCCACCTATGCGTGCTACCGTGAA	80	0.46 ± 0.08 μM	[70]
Di(2-ethylhexyl) phthalate (DEHP) CAS no.: 117-81-7 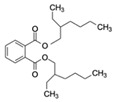	Plasticizer	ACGCATAGGGTGCGACCACATACGCCCCATGTATGTCCCTTGGTTGTGCCCTATGCGT	58	2.26 ± 0.06 nM	[71]
Acyclovir (ACV) CAS no.: 59277-89-3 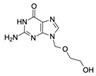	Aminoglycoside Antibiotics	TGAGCCCAAGCCCTGGTATGTGAAAACATACTAGACGTGGCTATGTATTTTTAAATCAATGGCAGGTCTACTTTGGGATC	80	32.67 ± 4.127 nM	[72]
Glutamate CAS no.: 56-86-0 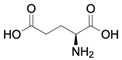	Excitatory neurotransmitter	GCATCAGTCCACTCGTGAGGTCGACTGATGAGGCTCGATCAGGAGCGCCGCTCGATCGCACTTTCACAGGATAGTAGTTGGTAGCGACCTCTGCTAGA	98	12 ± 6 μM	[73]
Famciclovir (FCV) CAS no.: 104227-87-4 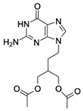	Aminoglycoside Antibiotics	TGAGCCCAAGCCCTGGTATGTGAAAACATACTAGACGTGGCTATGTATTTTTAAATCAATGGCAGGTCTACTTTGGGATC	80	47.35 ± 10.42 nM	[72]
Ganciclovir (GCV) CAS no.: 82410-32-0 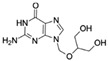	Aminoglycoside Antibiotics	TGAGCCCAAGCCCTGGTATGTGAAAACATACTAGACGTGGCTATGTATTTTTAAATCAATGGCAGGTCTACTTTGGGATC	80	47.91 ± 13.47 nM	[72]
Penciclovir (PCV) CAS no.: 39809-25-1 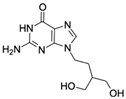	Aminoglycoside Antibiotics	TGAGCCCAAGCCCTGGTATGTGAAAACATACTAGACGTGGCTATGTATTTTTAAATCAATGGCAGGTCTACTTTGGGATC	80	33.29 ± 5.851 nM	[72]
Levamisole (LEV) CAS no.: 14769-73-4 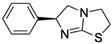	Veterinary drug	AATCAAACGCTAAGGTCAAGGGAGAGTGCACCCATTCTTGGGGCCCCGGGCCAGCCCCGACACGCCGCCGAAGCTTGGTACCCGTATCGT	90	66.15 ± 11.86 nM	[74]
Valaciclovir (VACV) CAS no.: 124832-26-4 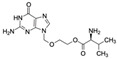	Aminoglycoside Antibiotics	TGAGCCCAAGCCCTGGTATGTGAAAACATACTAGACGTGGCTATGTATTTTTAAATCAATGGCAGGTCTACTTTGGGATC	80	44.26 ± 6.744 nM	[72]
Tetrodotoxin CAS no.: 4368-28-9 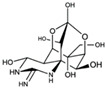	Toxin	ATACCAGCTTATTCAATTTAATGCGGGGTGAGGCTCAATCAAGGAAAGATATAAGTAAGCAAAAAGGTCAAACAAGGGCGAGATAGTAAGTGCAATCT	98	7 ± 1 nM	[43]
Ribavirin CAS no.: 36791-04-5 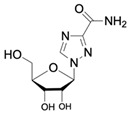	Antiviral agent	AAAGTAATGCCCGGTAGTTATTCAAAGATGAGTAGGAAAAGA	42	61.19 ± 21.48 nM	[45]
Gymnodimine-A CAS no.: 173792-58-0 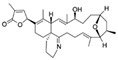	Toxin	GCGACCGAAGTGAGGCTCGATCCAAGGTGGACGGGAGGTTGGATTGTGCGTG	52	34.5 ± 1.72 nM	[75]
λ-cyhalothrin CAS no.: 91456-08-6 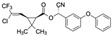	Pesticide	AGGGGAAGCACGGGCGGGCG	20	10.27 ± 1.33 nM	[76]
Malachite green (MG) CAS no.: 569-64-2 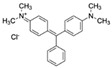	Veterinary drug	(1) CGCAGCGCGGCAGACAGTCAGGCTCAGCACGTGGCA	36	102.46 μM	[77]
		(2) CACTCCACGCATAGGGACGCGAATTGCGGACCTATGTGTGGTGTG	45	2.3 ± 0.2 μM	[78]
Leucomalachite green (LMG) CAS no.: 129-73-7 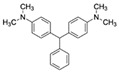	Antimicrobial	CACTCCACGCATAGGGACGCGAATTGCGGACCTATGTGTGGTGTG	45	8.2 ± 1.2 μM	[78]
Phorate CAS no.: 298-02-2 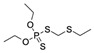	Organothiophosphate insecticide	AAGCTTGCTTTATAGCCTGCAGCGATTCTTGATCGGAAAAGGCTGAGAGCTACGC	55	1.11 μM	[79]
Profenofos CAS no.: 41198-08-7 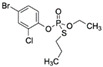	Organothiophosphate insecticide	AAGCTTGCTTTATAGCCTGCAGCGATTCTTGATCGGAAAAGGCTGAGAGCTACGC	55	1 μM	[79]
Isocarbophos CAS no.: 24353-61-5 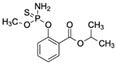	Organothiophosphate insecticide	AAGCTTGCTTTATAGCCTGCAGCGATTCTTGATCGGAAAAGGCTGAGAGCTACGC	55	0.83 μM	[79]
Omethoate CAS no.: 1113-02-6 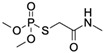	Organothiophosphate insecticide	AAGCTTTTTTGACTGACTGCAGCGATTCTTGATCGCCACGGTCTGGAAAAAGAG	54	2 μM	[79]
Cortisol CAS no.: 50-23-7 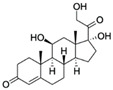	Steroid hormone	GGAATGGATCCACATCCATGGATGGGCAATGCGGGGTGGAGAATGGTTGCCGCA CTTCGGCTTCACTGCAGA CTTGACGAAGCTT	85	6.9 ± 2.8 nM	[80]
Tobramycin CAS no.: 32986-56-4 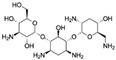	Aminoglycoside antibiotic	CCATGATTCAACTTTACTGGTCTTGTCTTGGCTAGTCGTGTGTCATTCCCGTAAGGG	57	200 nM	[47]
Clenbuterol hydrochloride (CLB) CAS no.: 21898-19-1 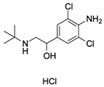	beta-adrenergic agonist	AGCAGCACAGAGGTCAGATGTCATCTGAAGTGAATGAAGGTAAACATTATTTCATTAACACCTATGCGTGCTACCGTGAA	80	76.61 ± 12.7 nM	[81]
Ractopamine CAS no.: 97825-25-7 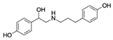	Veterinary drug	AGCAGCACAGAGGTCAGATGGTCTCTACTAAAAGTTTTGATCATACCGTTCACTAATTGACCTATGCGTGCTACCGTGAA	80	54.22 ± 8.02 nM	[82]
Atrazine CAS no.: 1912-24-9 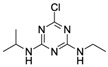	Herbicide	TGTACCGTCTGAGCGATTCGTACTTTATTCGGGAAGGGTATCAGCGGGGTTCAACAAGCCAGTCAGTCAGTGTTAAGGAGTGC	83	3.7 nM	[83]
Zearalenone CAS no.: 17924-92-4 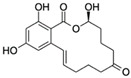	Mycotoxin	ATACCAGCTTATTCAATTCTACCAGCTTTGAGGCTCGATCCAGCTTATTCAATTATACCAGCTTATTCAATTATACCAGCACAATCGTAATCAGTTAG	98	15.2 ± 3.4 nM	[84]
Fenitrothion CAS no.: 122-14-5 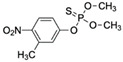	Phosphorothioate insecticide	CTCTCGGGACGACGGGCCGAGTAGTCTCCACGATTGATCGGAAGTCGTCCC	51	33.57 nM	[85]
Malathion CAS no.: 121-75-5 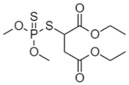	Organophosphate insecticide	GGGATACAGGTAGTATGGCATGTGCTAGCGGTTGCA	36	22.56 nM	[86]
Fipronil CAS no.: 120068-37-3 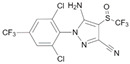	Insecticide	ACGACAGATAGTGTGTACATGAAGGGTCGT	30	15 nM	[87]
Diazinon CAS no.: 333-41-5 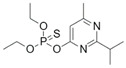	Organophosphate insecticide	TTCCGATCAATCGTGGAGACTACTCGGCCC	30	4.571 ± 0.714 µM	[88]
Ethyl carbamate (EC) CAS no.: 51-79-6 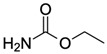	Organic compound	GGGGGCACGGGAGGT	15	17.97 ± 0.98 nM	[89]
3,4-methylenedioxypyrov-alerone (MDPV) CAS no.: 687603-66-3 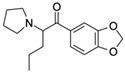	Synthetic cathinone	CTTACGACTCAGGCATTTTGCCGGGTAACGAAGTTACTGTCGTAAG	46	6.1 ± 0.2 μM	[90]
Dichlorodiphenyltrichloroethane (o,p’—DDT) CAS no.: 789-02-6 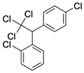	Insecticide	TCCAGCACTCCACGCATAACGAATTGTGCTCAATGCGCCCCTGCAGTGAATGTGGAATTTGTTATGCGTGCGACGGTGAA	80	412.3 ± 124.6 nM	[91]
Penicillin G CAS no.: 61-33-6 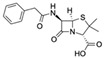	β-lactam antibiotic	GGGAGGACGAAGCGGAACGAGATGTAGATGAGGCTCGATCCGAATGCGTGACGTCTATCGGAATACTCGTTTTTACGCCTCAGAAGACACGCCCGACA	98	--	[21]

**Table 2:** Representative biosensors using aptamers against small molecule contaminants that are generated from Capture-SELEX.

Types	Aptamer Targets	Limit of Detection (LOD)	Detection Range	Ref.
Ion-sensitive field-effect transistor (ISFET)	Vanillin	0.155 μM	0.155–1.0 μM	[23]
	Furaneol	--	0.1–10 μM	[68]
Electrochemical impedance spectroscopy (EIS)	Penicillin G	0.00051 μM	1.2 nM–2.99 μM	[21]
	Di(2-ethylhexyl) phthalate (DEHP)	0.264 pM	--	[71]
	Glutamate	0.0013 pM	0.01 pM–1 nM	[73]
Fluorescent	Spermine	0.052 nM	0.1–20 nM	[44]
	Tyramine (TYR)	2.48 nM	3.64–728.97 nM	[109]
	β-phenethylamine	3.22 nM	4.13–825.22 nM	[109]
	Acyclovir	2.13 nM	8.88–444.03 nM	[72]
	Famciclovir	1.74 nM	6.22–311.2 nM	[72]
	Ganciclovir	2.08 nM	7.84–391.80 nM	[72]
	Penciclovir	1.97 nM	7.9–394.85 nM	[72]
	Valaciclovir	1.17 nM	6.17–308.32 nM	[72]
	Ribavirin	2.74 nM	4.09–204.75 nM	[45]
	Malachite Green	5.84 nM	4.69 nM–2.35 μM	[77]
	Cadmium ions (Cd(II))	40 nM	0–1000 nM	[111]
	Clenbuterol hydrochloride (CLB)	0.22 nM	0.32–159.44 nM	[81]
	Ractopamine	0.13 nM	0.33–331.79 nM	[82]
	Chlorpromazine	0.67 nM	1–100 nM	[69]
	Fenitrothion	14 nM	0–80 nM	[85]
	Malathion	6.08 nM	--	[86]
	Fipronil	3.4 nM	0–70 nM	[87]
	Diazinon	148 nM	0.1–25 μM	[88]
Lateral flow aptasensor (LFA)	Erythromycin	3 pM	1 pM–10 nM	[24]
	Ethyl carbamate	0.024 μM	0.11–0.67 μM	[89]
Colorimetric	Roxithromycin	0.077 μM	0–4.44 μM	[70]
	λ-cyhalothrin	41 nM	0.22–1.11 μM	[76]
	Zearalenone	12.5 nM	12.5–402.1 nM	[84]
	Levamisole	1.12 nM	1–200 nM	[74]
Biolayer interferometry (BLI)	Gymnodimine-A	6.21 nM	55–1400 nM	[75]

## Data Availability

Not applicable.
